# Supplementary material for: Seroprevalence and associated risk factors of Toxoplasma gondii infection among pregnant mother in Makassar, Indonesia
Source: PLoS One. 2021 Jun 4;16(6):e0245572. doi: 10.1371/journal.pone.0245572 (PMC8177418; doi:10.1371/journal.pone.0245572)
Supplement: Attachment — Submitted filename: Response to Reviewers.docx [file pone.0245572.s002.docx]

Makassar, March 16^th^ 2021

Dear Academic Editor and Reviewer (s),

We have made improvements to the manuscript by following the recommendations of the reviewers. These improvements are as follows:

1. Although we found some factors are associate with Toxoplasma gondii IgG positivity, we realize that the range of 95% confidence intervals are wide indicating the sample size is small. During Covid-19 pandemic, the number of pregnant women who came to the Primary Health Centers decrease 50-70 than in normal situation. Initially, we collected 200 samples but 16 samples had to be excluded due to insufficient serum and blood lysis. Unfortunately, when we decided to add more participants, our city was locked-down due to an increase in the number of Covid-19 cases. It is unknown when the pandemic will end and we worried if delayed too long the quality of existing serum decreases, we then analyzed the existing samples whose numbers were not too far from the minimum sample size determined by the Lemshow formulas (n=188). If the same studi is to be repeated, it is recommended that the number of samples be increased (line 252-261).
2. From previous studies, age is one of the factors that influence immune response to *Toxoplasma* infections. In our study, we have checked the possibility of age as a confounding factor. Using Levene's test we found p=0.387 indicated that the age of pregnant women in seropositive and seronegative are homogenous, therefore we did not include age as a confounding factor (line 171-174).
3. We have read the recommended paper and we found several information:
a. Paper from Nigeria written by Gyang, *et al.* conducted a study of seroprevalence and associated risk factors among primary schoolchildren. Among the risk factors tested including contact with cats, none showed statistical significance associated. The paper has a different object than us and did not explain about what kind of contact with cat it is, whether it is just stroking or sleeping together. However, in our paper, we have mentioned that contact with cat means not only physical contacts like stroking or carrying but also contact with body part of cat.
b. Another Research conducted in Swaziland by Liao, *et al*. explained that seropositivity among schoolchildren was around 8% positive for *Toxoplasma gondii*. However, this paper did not discuss the association of contact with cats with exposure to *Toxoplasma gondii*. They discussed about the risk factors like gender and age group among the children. However, we found another study carried out in Tunisia that investigated the association between contact with cat and seropositivity of *Toxoplasma gondii* in pregnant women. They found that contact with cat was independently associated with positivity of *Toxoplasma gondii* antibody.

The paper mentioned above were included in discussion (line 202-205)

4. During the interview, we have confirmed that the satay in this study refers to chicken satay and not beef / goat / buffalo satay or steak. People of Makassar tend to consume chicken satay rather than beef which mostly serves as a soup or other dish that is processed with long time cooking. Furthermore, Indonesian prefer to consume chicken satay that is not too dry (undercooked) to avoid tough meat and lack of taste. To ensure that chicken satay is undercook meat, three selling spots satay in different sub-districts in Makassar were surveyed and found that the length of burning time was vary between 3-6 minutes. The temperature of the satay once it is placed on the plate ranges from 40^o^C to 51^o^C and when the meat was cut open, the inside part was still juicy and reddish in colour indicated the chicken satay was not well-done or undercooked. A study done a few decades ago reported that *Toxoplasma* cysts and faecal toxoplasma were killed after exposure to a temperature of 55^o^C for 30 min and according to the central for disease control (CDC), to avoid infection with tissue cysts from *Toxoplasma*, poultry meat should be cooked at a minimum temperature of 74^o^C (line 229-237)

We hope that revision we propose meet the PLOS ONE’s publication criteria

Yours sincerely,

Sitti Wahyuni
